# Plain Talks on Medical Electricity and Batteries

**Published:** 1891-09

**Authors:** 


Plain Talks on Medical Electricity and Batteries. By Horatio R. Bigelow, M. D. Blakiston, Son & Co. Philadelphia, Pa.

This is a clever little monograph of 85 pages, and is intended more particularly for those who are not conversant with medical electricity. It is divided into four chapters, which include the following subject matter: First is the definitions of the electrical terms, such as electro-motor force, volts, resistance, ohms, intensity, ampere, physoilogical action of currents, etc. Second: Static electricity. Third: The galvanic current. 4th: The faradic or induced current; also, motor points and a therapeutic index. The book is practical, and deserves being read by all who are beginning the study of electricity.

